# Effect of Carbon Monoxide-Releasing Molecules II-liberated CO on Suppressing Inflammatory Response in Sepsis by Interfering with Nuclear Factor Kappa B Activation

**DOI:** 10.1371/journal.pone.0075840

**Published:** 2013-10-07

**Authors:** Weiting Qin, Jinli Zhang, Wanghui Lv, Xu Wang, Bingwei Sun

**Affiliations:** Department of Burn and Plastic Surgery, Affiliated Hospital, Jiangsu University, Zhenjiang, Jiangsu Province, China; Harvard Medical School, United States of America

## Abstract

Sepsis continues to be a challenge in clinic. The rates of mortality in sepsis patients remain high. The present study aimed to investigate the effects and the underlying mechanisms of carbon monoxide-releasing molecules II (CORM-2)-liberated CO on suppressing inflammatory response in sepsis. It was shown that treatment of septic mice with CORM-2 attenuated PMN accumulation, downregulated cytokines production, inhibited expressions of iNOS and NF-κB activity in the lung and liver. In parallel, CORM-2 prevented activation of NF-κB in LPS-stimulated HUVEC. This was accompanied by a decrease in ROS and NO production, expression of ICAM-1 and subsequent PMN adhesion to HUVEC. These findings demonstrated that CORM-released CO attenuates inflammatory responses by interfering with NF-κB activation and therefore decreasing the expression of ICAM-1 and NO production, attenuating the oxidative stress and inflammation in sepsis.

## Introduction

CLP (cecal ligation and puncture) may induce the activation of an inflammatory cascade, cause damage to multiple organs distant from the original burn wound and may lead to sepsis and multiple organ failure [1]–[4]. There have been several reports indicating that the inflammatory response syndrome and sepsis, which contributes to oxidative cell/tissue damage, might frequently be accompanied by leukocyte sequestration in many important organ systems in the body [5]. The increase of production of pro-inflammatory mediators such as interleukin (IL)-6 and tumor necrosis factor (TNF)-α is closely associated with activation of leukocytes and macrophages which were sequestrated in the tissue [6]–[8]. Leukocytes sequestration and their subsequent infiltration in organ tissue can cause leukocyte activation and contribute to vascular damage and the development of systemic inflammatory reaction. As the prerequisite, activation of leukocytes and endothelial cells results in aggregation of leukocytes, platelets and erythrocytes *in vivo*. This may favor disseminated intravascular coagulation and further multiple organ failure.

Carbon monoxide (CO) has long been known in biology and medicine as a toxic compound, due to its ability to bind hemoglobin with a much higher affinity than oxygen [9]. Evidence accumulated to date suggests that endogenous carbon monoxide (CO), a bi-product of inducible heme oxygenase (HO-1) can modulate inflammation, inhibits lipopolysaccharide (LPS)-induced production of cytokines both *in vivo* and *in vitro*, and consequently exhibits important cytoprotective function and anti-inflammatory properties [10]–[15]. Inhaled CO at concentrations of 250–500 parts per million (ppm) has also been shown to be beneficial in a number of lung injury models, including hyperoxic injury [16], [17], allergen-induced inflammation [18].

Recently, transition metal carbonyls have been identified as potential CO-releasing molecules (CO-RMs) with the potential to facilitate the pharmaceutical use of CO by delivering it to tissues and organs [19]. CO-RMs have been shown to act pharmacologically in rat aortic and cardiac tissue where liberation of CO produced vasorelaxant effects [20]–[23] and decreased myocardial ischemia–reperfusion damage [24], [25] in the absence of dramatic changes in blood carboxy-hemoglobin (COHb) levels. Our earlier studies first confirmed that CORM-released CO attenuated leukocytes sequestration in the liver, lung and small intestine of burn injury mice by interfering with nuclear factor-κB (NF-κB) activation [26]–[29].

On the basis of these data, the present study was, therefore, designed as a prospective laboratory experiment to investigate the effects of tricarbonyldichlororuthenium (II) dimer (CORM-2), one of the novel group of CO-RMs, on attenuation of leukocyte sequestration and decrease of inflammatory responses and oxidative stress in the organs of CLP-induced mice and LPS-induced HUVECs (human umbilical vein endothelial cells), and discuss the possible molecular mechanisms.

## Materials and Methods

### Ethics Statement

The Medical Ethical Committee of Jiangsu University approved the study. After written informed consent, blood specimens were obtained from healthy donors’ cubital veins. Consent for the use of these samples was given by the Medical Ethical Committee of Jiangsu University. The animal research was approved by the Council on Animal Care at Jiangsu University on the Protection and the Welfare of Animals and followed the National Institutes of Health guidelines for the care and use of experimental animals.

### Materials

Medium 199 (M199), fetal calf serum (FCS), penicillin, and streptomycin were purchased from GIBCO BRL (Gland Island, NY). Tricarbonyldichlororuthenium(II) dimer (CORM-2) was obtained from Sigma Aldrich and solubilized in dimethyl sulfoxide (DMSO) to obtain a 10 mM stock [27]. Inactive form (iCORM-2) of the compound (nega-tive control) was also used in some experiments and it was prepared as described previously [30]. LPS (Escherichia coli serotype 055:B5) was purchased from Sigma. Anti-ICAM-1 and Anti-iNOS polyclonal antibody was purchased from Transduction Laboratories (Lexington, KY). Anti-mouse IgG conjugated to horseradish peroxidase was purchased from Kirkegaard and Perry Laboratories (Gaithersburg, MD).

### Animals and Cecal Ligation and Puncture (CLP)

The C57BL/6 mice (male, N = 44; body weight 20±2 g) were fed a standard laboratory diet and water ad libitum. Mice were divided to four groups. Mice in sham group (n = 11) were underwent sham procedure, whereas mice in CLP group (n = 11) received cecal ligation and puncture, mice in CORM-2 group (n = 11) and iCORM group (n = 11) subjected to the same injury with immediate administration of CORM-2 (8 mg/kg, i.v.) and iCORM-2 (8 mg/kg, i.v.), respectively. The concentration of CORM-2 used in the present study was based on a previous report in our lab [26]–[29].

Mice were anesthetized with 2% isoflurane in oxygen via a facemask. A 2-cm midline incision was made through the abdominal wall; the cecum was identified and ligated with a 3-0 silk tie 1 cm from the tip. A single puncture of the cecal wall was performed with a 20-gauge needle. The cecum was lightly squeezed to express a small amount of stool from the puncture site to assure a full-thickness perforation. Inspection of mice at various intervals after CLP did not reveal evidence of bowel obstruction. The cecum was returned to the abdominal cavity, and the incision was closed with surgiclips. Sham mice underwent anesthesia and midline laparotomy. The cecum was exteriorized and returned to the abdomen, and the wound was closed with surgiclips. The animals were sacrificed at 24 h after experimental manipulation.

### Measurement of Mice Survival Rate

A total of 48 C57BL/6 male mice (aged 6–8 weeks and weighing 20±2 g) were fed in laboratory for 1 week. The mice were divided into four groups (n = 12) and treated with or without CORM-2 as described above. Mouse survival was monitored six times daily for up to 72 h.

### Measurement of TNF-α and IL-6 Levels in Serum

Blood samples were obtained by cardiac puncture of the left ventricle. The samples were stored in serum tubes (Capiject, Terumo Medical Corporation, USA) and immediately centrifuged at 6500 r/min for 5 min. Concentrations of TNF-α and IL-6 levels in serum were assayed by enzyme-linked immunosorbent assay kits (Genzyme, Corp., Cambridge, MA) following the manufacturer’s instructions.

### MPO Activity

MPO activity as an assessment of neutrophil influx was measured according to established protocols[31]–[33].In brief, tissue was homogenized in 0.5 ml of 50 mM potassium phosphate buffer (pH 7.4) and centrifuged at 10,000 *g* at 4°C for 30 min. The remaining pellet was resuspended in 0.5 ml of 50 mM potassium buffer pH 6.0 with 0.5% hexadecyltrimethylammonium bromide, sonicated on ice, and then centrifuged at 12,000 *g* at 4°C for 10 min. Supernatants were then assayed at a 1∶20 dilution in reaction buffer containing 50 mM PB, 530 mM o-dianisidine, and 20 mM H_2_O_2_ solution. One unit of enzyme activity was defined as the amount of MPO present that caused a change in absorbance measured at 460 nm for 3 min. MPO activity was expressed as U/g tissue.

### Isolation and Culture of Human Umbilical Vein Endothelial Cells (HUVECs)

Human umbilical vein endothelial cells (HUVECs) were harvested from the fresh human umbilical vein of newborns by collagenase treatment (Worthington Biochem, Freehold, NJ) as previously described [34]. The cells were grown in medium 199 (M199; GIBCO, Burlington, Canada) supplemented with 10% heat-inactivated FCS (Intergen, Purchase, NY), 2.4 mg/l thymidine (Sigma Chemical, Oakville, Canada), 10 IU/ml heparin sodium, antibiotics (100 U/ml penicillin and 100 µg/ml streptomycin; GIBCO), 1.5 µg/ml fungizone (GIBCO),and 80 µg/ml endothelial mitogen (Biomedical chnologies, Stoughten, MA). The cell cultures were incubated in room air with 5% CO2 at 37°C and 95% humidity and were expanded by brief trypsinization with 0.25% trypsin in PBS containing 0.025% EDTA. The experiments were conducted on passage 3 HUVECs. Cells were stimulated with LPS (10 µg/ml) for 4 h. The cells and medium were harvested separately.

### PMN Adhesion Assays

Human neutrophilic PMN were isolated from the venous blood of healthy adults using standard dextran sedimentation and gradient separation on Histopaque-1077. This procedure yields a PMN population that is 95–98% viable (trypan blue exclusion) and 98% pure (acetic acid-crystal violet staining). For the static adhesion assay, isolated neutrophils were suspended in PBS buffer and radiolabeled by incubating the cells at 5×10^7^ cells/ml with 50 µCi Na^51^CrO_4_/ml PMN suspension at 37°C for 60 min. The cells were then washed with cold PBS to remove unincorporated radioactivity. Radiolabeled PMN (5×10^5^/well) were added to HUVEC monolayers grown in 48-well plates (Costar), and 30 min later the percentage of added PMN that remained adherent after a wash procedure was quantitated as follows: %PMN adherence = lysate (cpm)/[supernatant (cpm)+wash (cpm)+lysate (cpm)], where cpm is counts per minute.

### Oxidant Production

Oxidant production within HUVECs was assessed by measuring the oxidation of intracellular dihydrorhodamine 123 (DHR 123; Molecular Probes, Inc.), an oxidant-sensitive fluorochrome, as described previously [35]. Briefly, the cells were treated with DHR 123 (5 mM) for 1 h before being subjected to LPS stimulation. After LPS stimulation, the cells were washed with PBS, lysed, and DHR 123 oxidation was assessed spectrofluorometrically at excitation and emission wavelengths of 502 and 523 nm, respectively.

### Nitric Oxide Production

NO production by HUVECs was assessed by measuring the fluorescence of 4-amino-5-methylamino-2′,7′-difluorofluorescein diacetate (DAF-FM diacetate), a specific NO probe (Molecular Probes, Inc.) [36]. Briefly, DAF-FM diacetate (10 mM) in M199 was added to the HUVEC 1 h before the LPS stimulation. After LPS stimulation, the HUVEC and supernatants were collected and analyzed spectrofluorometrically at excitation and emission wavelengths of 495 and 515 nm, respectively.

### SDS-polyacrylamide Gel Electrophoresis and Western Blotting

SDS-polyacrylamide gel electrophoresis and Western Blotting were performed as described previously [28], [29]. Samples (10 µg of protein) were subjected to electrophoresis on 7% SDS-polyacrylamide gels, with the use of the discontinuous system and transferred onto nitrocellulose membranes. The membranes were probed with anti-ICAM-1 monoclonal antibody (1∶2500). anti-iNOS monoclonal antibody (1∶2000) followed by anti-mouse IgG conjugated to horseradish peroxidase (1∶2500) as a secondary antibody. The bands were visualized by the use of ECL reagent and Hyperfilm ECL (Amersham, Arlington Heights, IL) as described by the manufacturer. Films were scanned using a flatbed scanner and the bands were quantified using Basic Quantifier software (Bio Image, Ann Arbor, MI), an image analysis program, on computer.

### Nuclear Protein Extraction and Electrophoretic Mobility Shift Assay (EMSA)

Nuclear protein from whole tissue (medial lobe of liver) was extracted using the methods as described Previously [28], [29]. Nuclear protein was extracted from HUVECs as previously described [34]. Cells were grown to confluence in Petri-dish, scraped, washed with cold PBS, and incubated in 150 µl of buffer E(+) (0.3% Nonidet P-40, 10 mM Tris (pH 8.0), 60 mM NaCl, 1 mM EDTA, 0.5 mM dithiothreitol (DTT), 1 µg/ml aprotinin, 1 µg/ml leupeptin, and 1 mM phenylmethylsulfonyl fluoride ) for 5 min on ice. Samples were centrifuged at 4°C for 5 min at 500 g. The supernatant was then removed, and the pellets (nuclei) were resuspended in 150 µl of buffer E (10 mM Tris (pH 8.0), 60 mM NaCl, 1 mM EDTA, and 0.5 mM DTT) and centrifuged at 500 g for 5 min at 4°C. The nuclei were then extracted in 30–50 µl of buffer E(c)(20 mM HEPES, 0.75 mM spermidine, 0.15 mM spermine, 0.2 mM EDTA, 2 mM EGTA, 2 mM DTT, 20% glycerol, and 1 mM PMSF (4°C) in the presence of 0.4 MnaCl) and were incubated on ice for 20 min. Finally, the samples were centrifuged for 10 min at 500 g (4°C), and the supernatants were collected and saved as the nuclear protein fraction. Samples were stored at −80°C. The double-stranded oligonucleotide containing consensus (58-AGGGACTTCCGCTGGGGACTTTCC-38) binding sites for NF-κB (synthesized on site; Beckman-Oligo 1000 M DNA synthesizer) were endlabeled with [**r**-^32^P]ATP (Amersham) by using T4-polynucleotide kinase (MBI Fermentas, Flamborough, ON). One picomole of the labeled oligonucleotide was incubated with 5 µg of nuclear extract protein in the presence or absence of 50× excess of cold oligonucleotide. Samples were incubated for 30 min at room temperature and then run through a 4% nondenaturing polyacrylamide gel at 280V for 45–60 min. The gel was dried and then exposed to X-ray film (Kodak) in cassettes for 2–4 h at −80°C with intensifying screens. Cold competition oligonucleotides were used to determine the specificity of the EMSA experiments for measuring NF-κB binding activities.

### Cell ELISA

For assessment of ICAM-1 surface expression level, an ELISA was performed [37], [38] on HUVECs grown in 96-well cell culture plates (Corning). HUVECs were fixed in 4% paraformaldehyde at 4°C for 30 min. The cells were then washed two times with cold PBS and were incubated with the mouse primary monoclonal antibody (MAb) against human ICAM-1 (Dako) at a concentration of 10 µg/ml for 1 h at room temperature. After this treatment, immunocytochemical staining of HUVEC monolayers was performed using an avidin-biotin-conjugated peroxidase mouse IgG staining kit (Vectastain), and MAb binding was subsequently quantified with a microplate reader (model 3550-UV; Bio- Rad) at 450-nm wavelength.

### Statistical Analysis

All of the values are presented as means ± SE. Statistical analysis was performed with the use of ANOVA and Student’s *t*-test for the comparisons. A value of *P*<0.05 was considered to be statistically significant.

## Results

### Effect of CORM-2 on CLP-induced Mouse Survival Rate

**Figure 1:**
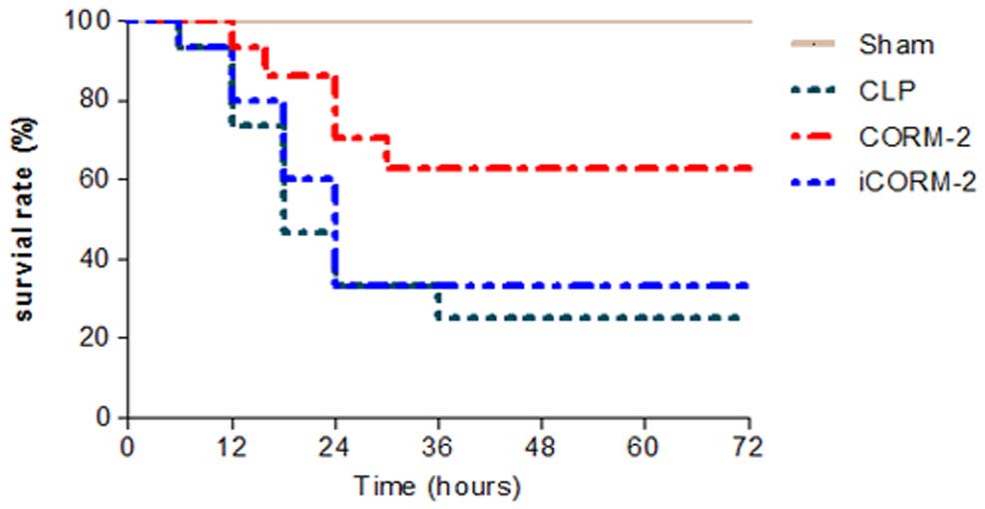
Effect of CORM-2 on the CLP-induced septic mice survival rate. CLP-induced mice were treated with or without CORM-2. Mouse survival was monitored for up to 72 h six times daily. Mice in sham group presented a 100% survival rate throughout the analyzed period of 72 hours. Mice in the CLP group began to die about 10 h and 75% death at 36 h. In contrast, the survival rates tended to improve with 62% survival at 36 h in the CLP+CORM-2 group treated with 8 mg/kg CORM-2. The bar graph illustrates the survival rate for each group at 72 h after the surgical procedure. (n = 12 animals per group).

CLP-induced mice were treated with or without CORM-2. Mouse survival was monitored for up to 72 h six times daily. Mice in the CLP group began to die about 10 h and 75% death at 36 h. In contrast, the survival rates tended to improve with 62% survival at 36 h in the CLP+CORM-2 group treated with 8 mg/kg CORM-2 (Figure 1).

### Effect of CORM-2 on Serum Cytokine Levels in Septic Mice

**Figure 2:**
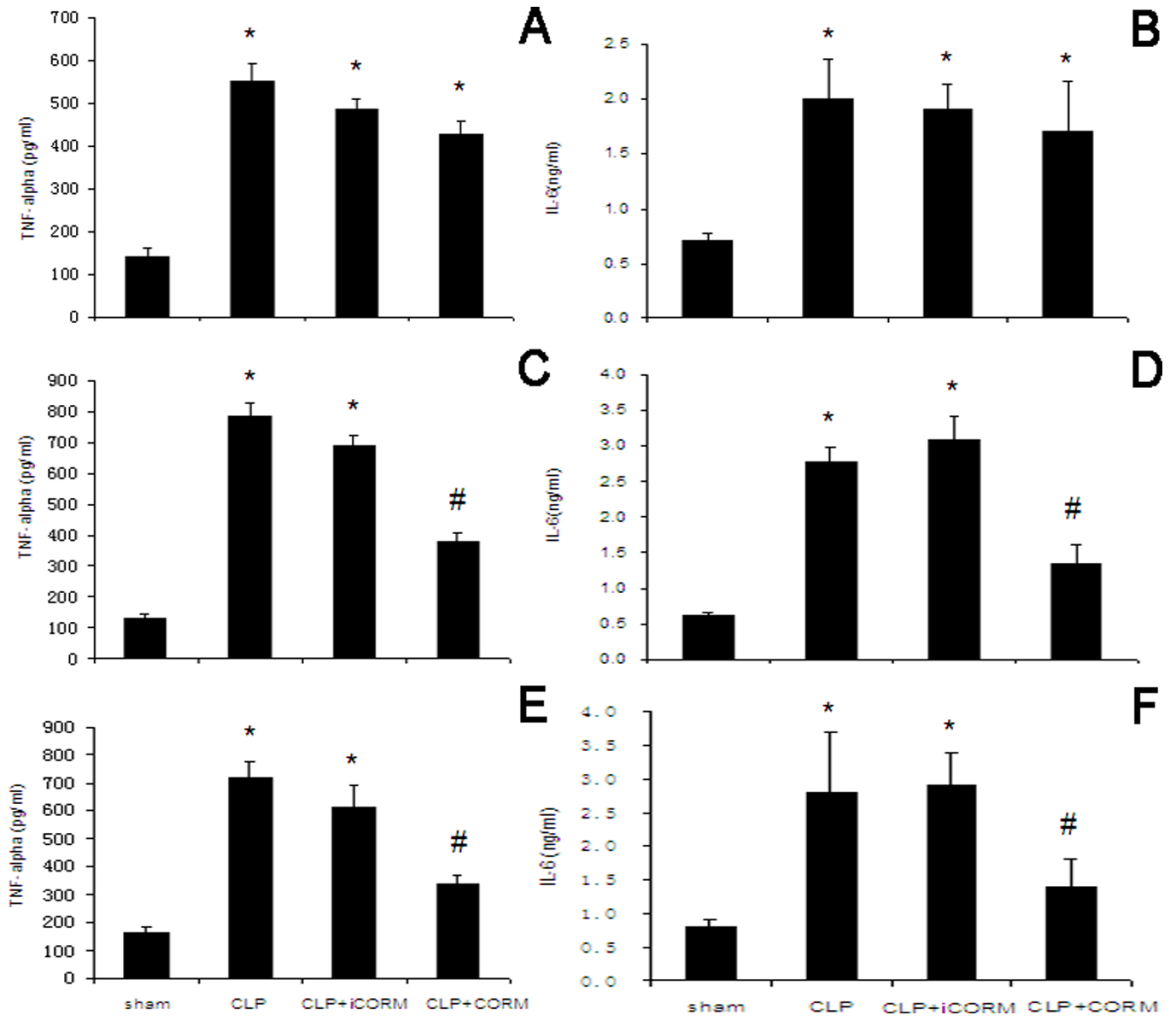
Effect of CORM-2 on TNF-α and IL-6 levels in serum of septic mice. Mice were challenged with CLP and treated with CORM-2 or iCORM-2. Blood samples were obtained by cardiac puncture of the left ventricle. Concentrations of TNF-α and IL-6 levels were assayed by enzyme-linked immunosorbent assay kits. At 6 after CLP, TNF-α and IL-6 levels in serum of the CLP-challenged mice were markedly increased compared to the sham mice. This increase wasn’t abolished by administration of CORM-2 (A, B). However, the elevation in serum levels of TNF-α and IL-6 was significantly abolished by CORM-2 treatment at 12 and 24 h (C, D, E and F). Data are expressed as mean ± SE of three experiments. *P<0.05 compared to sham group; #*P*<0.05 compared to CLP group.

At 6 after CLP, TNF-α and IL-6 levels in serum of the CLP-challenged mice were markedly increased compared to the sham mice. This increase wasn’t abolished by administration of CORM-2 (Figure 2A and B). However, the elevation in serum levels of TNF-α and IL-6 was significantly abolished by CORM-2 treatment at 12 and 24 h (Figure 2C, D, E and F).

### Effect of CORM-2 on Organs MPO Activity in Septic Mice

**Figure 3:**
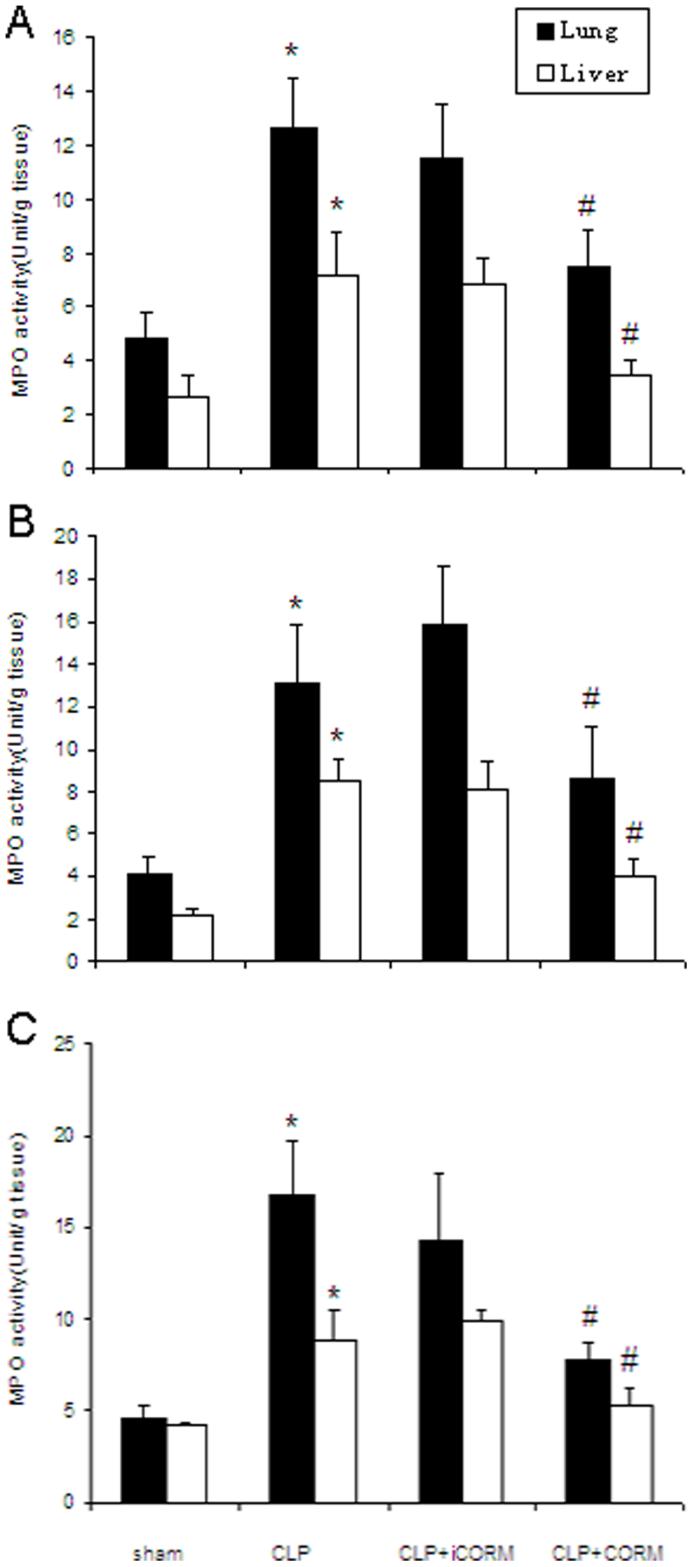
Effects of CORM-2 on MPO activity in the Lung and Liver of septic mice. Mice were challenged with CLP and treated with CORM-2 as described in Figure 2. MPO activities in the lung and liver were assessed at 6, 12 and 24 h after CLP. MPO activity in organs obtained from CLP-induced mice was markedly increased compared to sham, while it significantly decreased by treatment with CORM-2 (A, B, C). Results are mean ± SE of three experiments, *P<0.01 compared to sham mice. #*P*<0.05 compared to CLP mice.

To determine whether CLP-induced increase in PMN accumulation in the lung and liver was effectively prevented by CORM-2, the activity of MPO, an enzyme in azurophilic granules of neutrophils, was assessed. Extracts of the organ samples were examined for content of MPO at 6, 12 and 24 h after CLP injury. MPO activity in organs obtained from CLP-induced mice was markedly increased compared to sham (*P*<0.01), while it significantly decreased by treatment with CORM-2 (Figure 3A, B and C).

### Effect of CORM-2 on Expression of iNOS in the Lung and Liver in Septic Mice

**Figure 4:**
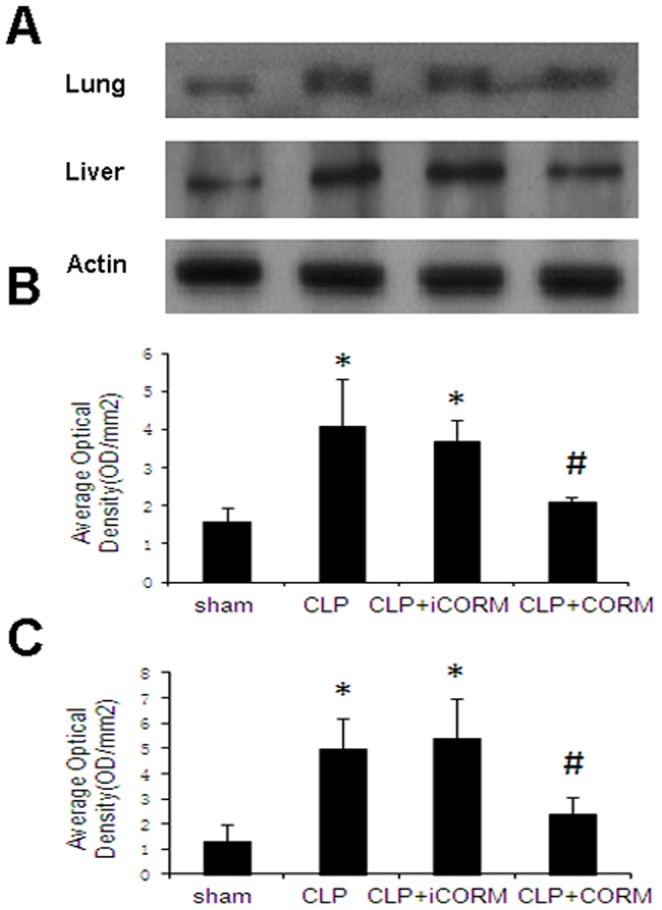
Effects of CORM-2 on protein expression of iNOS in the liver and lung of septic mice. Mice were challenged with CLP and treated with CORM-2 as described in Figure 2. Protein expression of iNOS in lung and liver was performed by western blotting at 24 h after CLP. A representative image is shown in A, the average optical density in three experiments are shown in B (lung) and C (liver), respectively. Results are mean ± SE. *P<0.01 compared to sham mice. #*P*<0.05 compared to CLP mice.

At 24 h after CLP induction, the expression of iNOS in lung and liver tissues significantly increased compared to the sham animals. *In vivo* administration of CORM-2 (8 mg/kg, i.v.), expression of iNOS was significantly decreased (Figure 4).

### Effect of CORM-2 on NF-κB Activity in the Lung and Liver in Septic Mice

**Figure 5:**
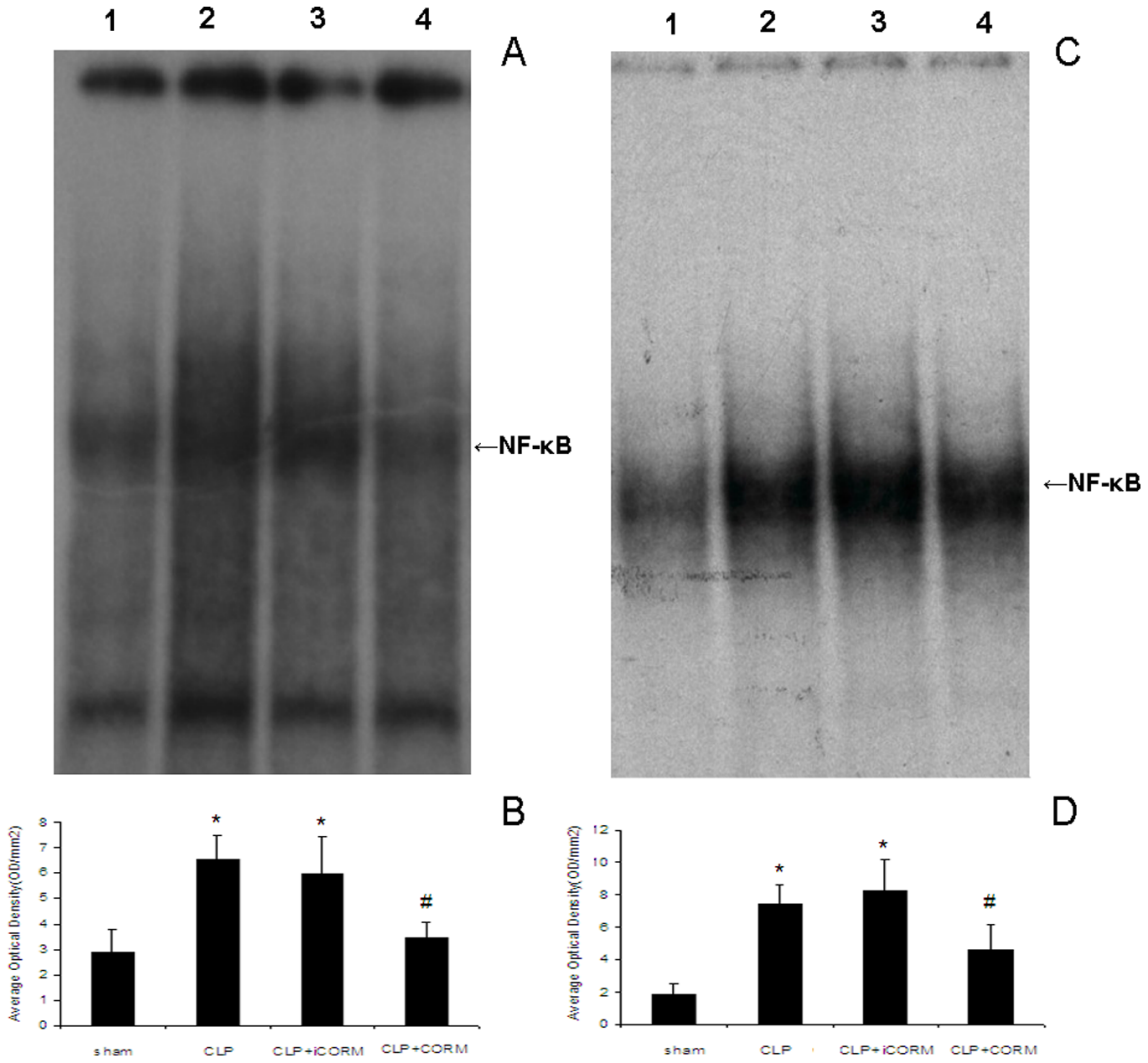
Effects of CORM-2 on NF-κB activation in the lung and liver of septic mice. Mice were challenged with CLP and treated with CORM-2 as described in Figure 2. Measurement of NF-κB activities were performed by mobility shift assay (EMSA) with ^32^ P-labeled NF-κB probe. The representative images are shown in A (lung) and C (liver), the average optical density in three experiments are shown in B (lung) and D (liver), respectively. All values are expressed as mean ± SE (*n* = 4). *p<0.05 compared to sham, #*p*<0.05 compared to CLP. Lane 1, sham; lane 2, CLP; Lane 3, CLP+iCORM; Lane 4, CLP+CORM.

Binding activities of nuclear protein to the radiolabeled consensus binding sequences of NF-κB was assessed by EMSA. At 24 h after CLP induction, the NF-κB activation in lung and liver was markedly increased and this activity was inhibited by *in vivo* administration of CORM-2 (8 mg/kg i.v.) (Figure 5).

### Effect of CORM-2 on Intracellular Production of ROS and NO in HUVECs Stimulated by LPS

**Figure 6:**
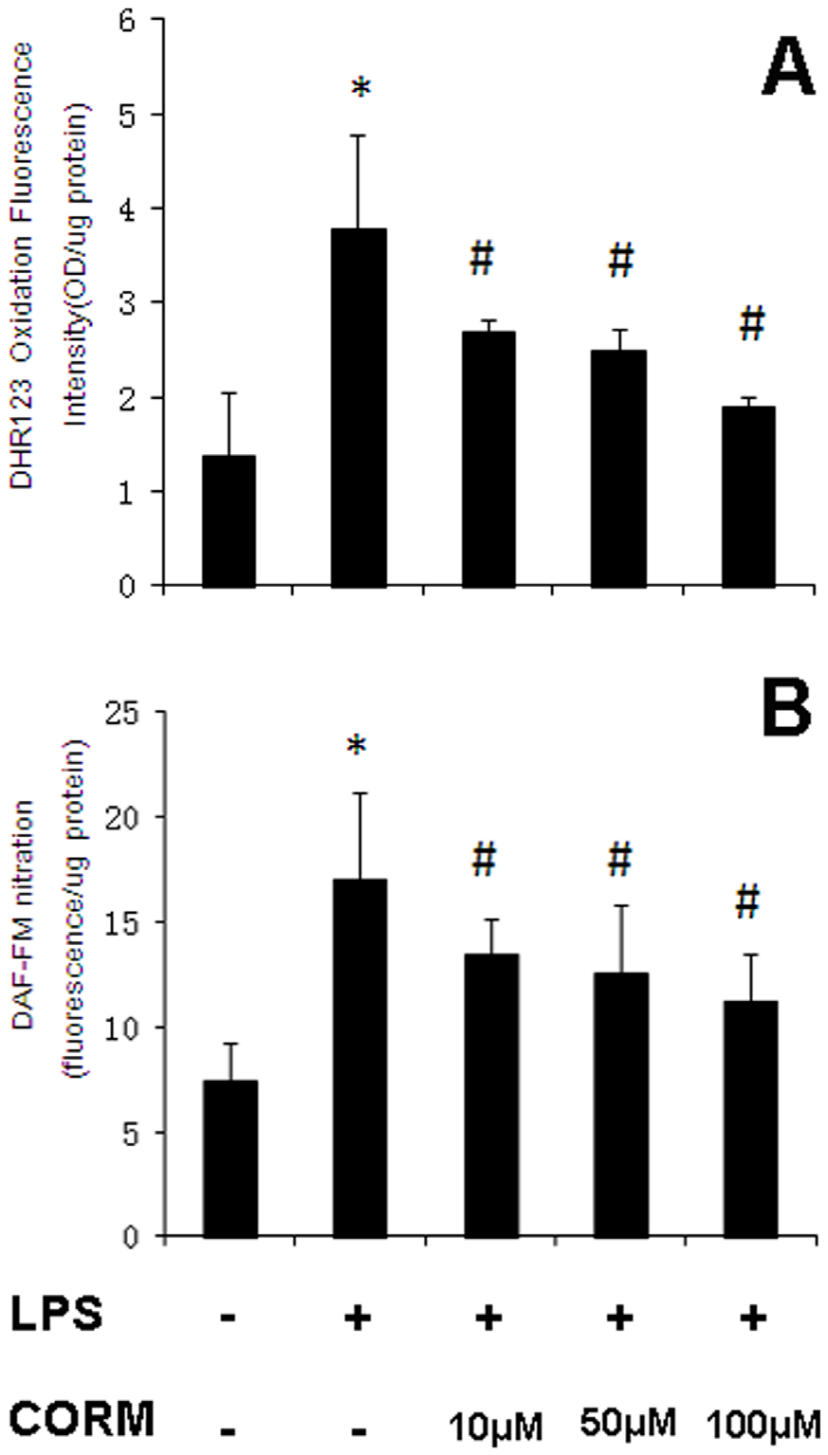
Effects of CORM-2 on intracellular production of ROS and NO in LPS-stimulated HUVECs. HUVECs were grown to confluence in 48-well cell culture plates and loaded with dihydrorhodamine 123 (DHR 123) or diaminofluorescein-FM (DAF-FM) for 1 h. Subsequently, HUVECs were stimulated with LPS (10 µg/ml) for 4 h in the presence or absence of CORM-2 (10, 50, 100 µM). Oxidative stress (DHR123 oxidation) (A) and NO production (DAF-FM nitration) (B) were assessed. All values are expressed as mean ± SE (*n* = 4). *p<0.05 compared to control, #*p*<0.05 compared to LPS. Note that LPS-induced oxidative stress and NO production were inhibited by CORM-2 in a dose-dependent manner.

As shown in Figure 6A, the LPS stimulation resulted in a significant increase in HUVECs oxidant production. Treated HUVECs with different concentration of CORM-2 induced less DHR oxidation compared to the LPS in a concentration dependent manner. As shown in Figure 6B, HUVECs produced significantly more NO during the LPS stimulation as compared to control. HUVECs significantly decreased their NO production after treatment with CORM-2 in a concentration dependent manner.

### Effect of CORM-2 on ICAM-1 Expression in LPS-stimulated HUVECs (Cell ELISA and Western Blotting)

**Figure 7:**
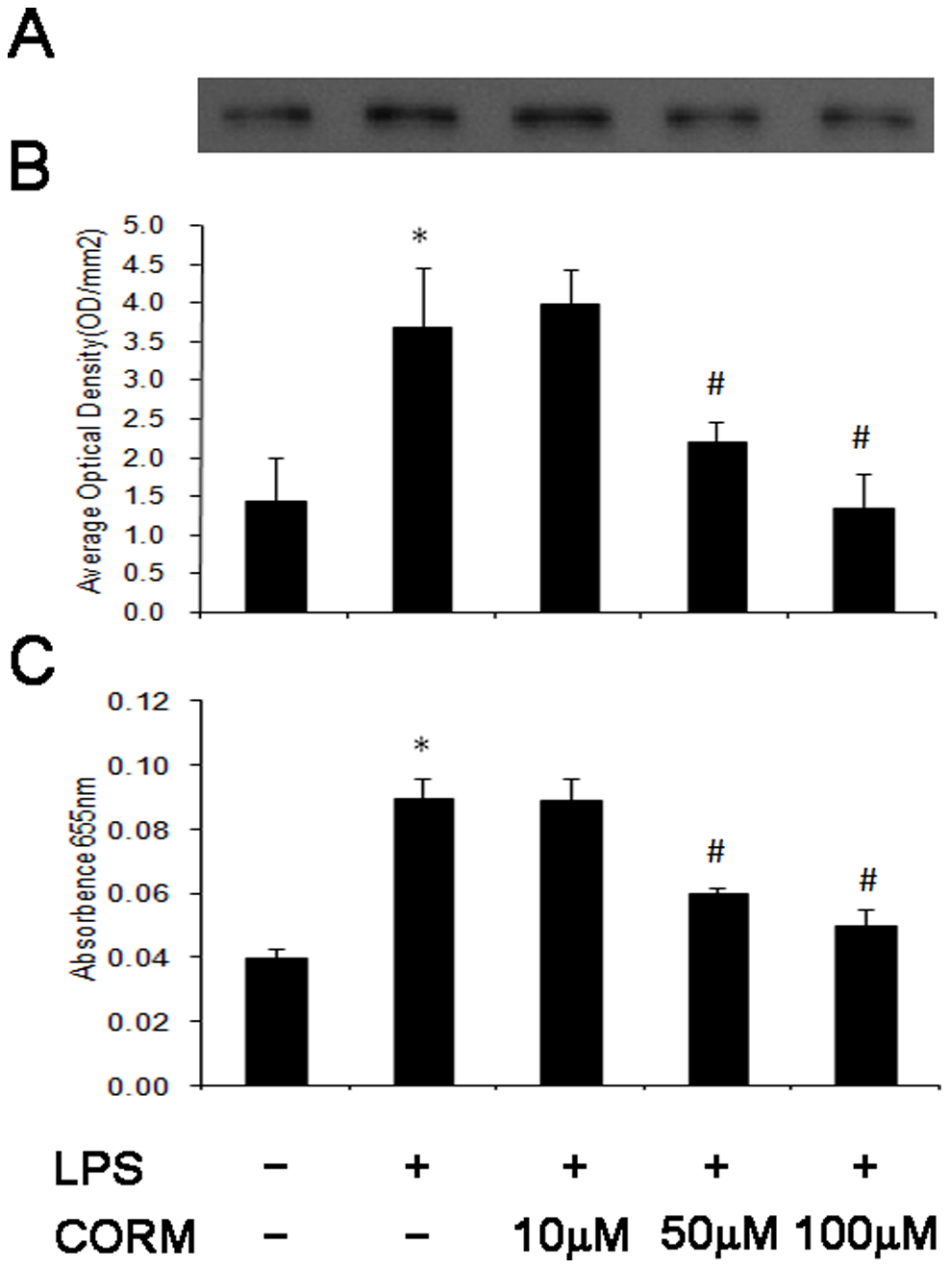
Effects of CORM-2 on ICAM-1 expression in LPS-stimulated HUVECs. HUVECs were grown to confluence in 48-well cell culture plates and were stimulated with LPS (10 µg/ml) for 4 h in the presence or absence of CORM-2 (10, 50, 100 µM). The expression of ICAM-1 were measured by western (A) and ELISA (C). A representative image is shown in A, the average optical density in three experiments are shown in B. All values are expressed as mean ± SE (*n* = 4). *p<0.05 compared to control, #*p*<0.05 compared to LPS. Note that LPS-induced expression of ICAM-1 was downregulated by CORM-2 in a dose-dependent manner.

At 4 h after LPS stimulation, the expression of ICAM-1 in HUVECs significantly increased compared to the control. At the present of CORM-2 (10, 50 and 100 µM), expression of ICAM-1 (Figure 7A, western blotting and 7C, ELISA) was significantly decreased.

### Effect of CORM-2 on Activities of NF-κB in LPS-challenged HUVECs

**Figure 8:**
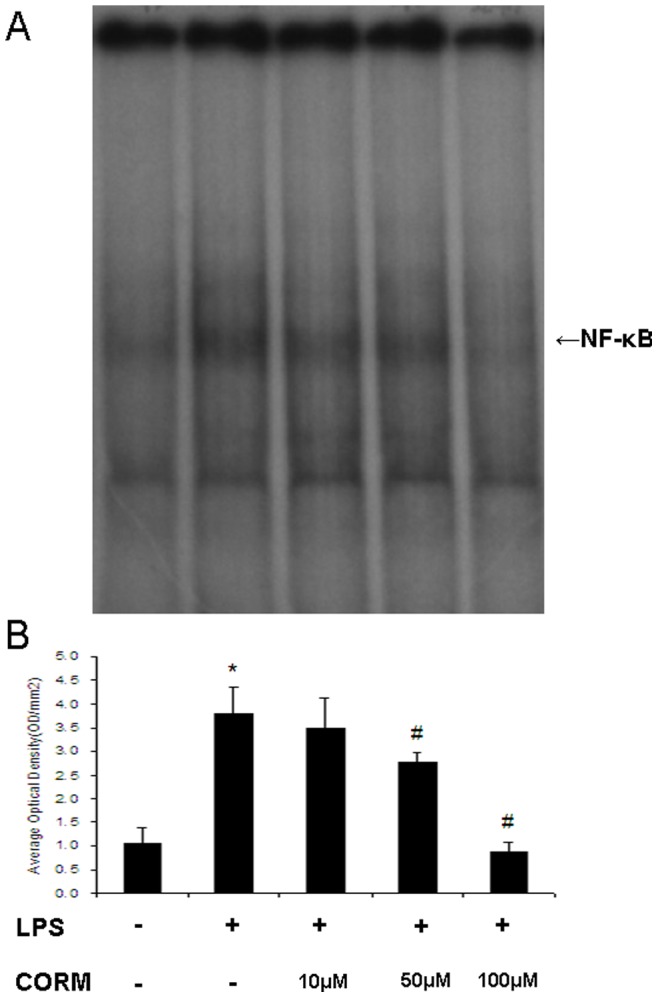
Effects of CORM-2 on NF-κB activation in LPS stimulated-HUVECs. HUVECs were grown to confluence in 48-well cell culture plates and were stimulated with LPS (10 µg/ml) for 4 h in the presence or absence of CORM-2 (10, 50, 100 µM). The NF-κB activation in HUVECs was measured by EMSA. The NF-κB activation was markedly increased in LPS-stimulated HUVECs and this increase was inhibited by administration of CORM-2 in a concentration dependent manner. A representative image is shown in A, the average optical density in three experiments are shown in B. All values are expressed as mean ± SE (*n* = 4). *p<0.05 compared to control, #*p*<0.05 compared to LPS.

Binding activities of nuclear protein to the radiolabeled consensus binding sequences of NF-κB was assessed by EMSA. At 4 h after LPS stimulation, the NF-κB activation in HUVECs was markedly increased and this activity was inhibited by administration of CORM-2 in a concentration dependent manner (Figure 8).

### Effect of CORM-2 on PMN Adhesion to HUVECs Stimulated by LPS

**Figure 9:**
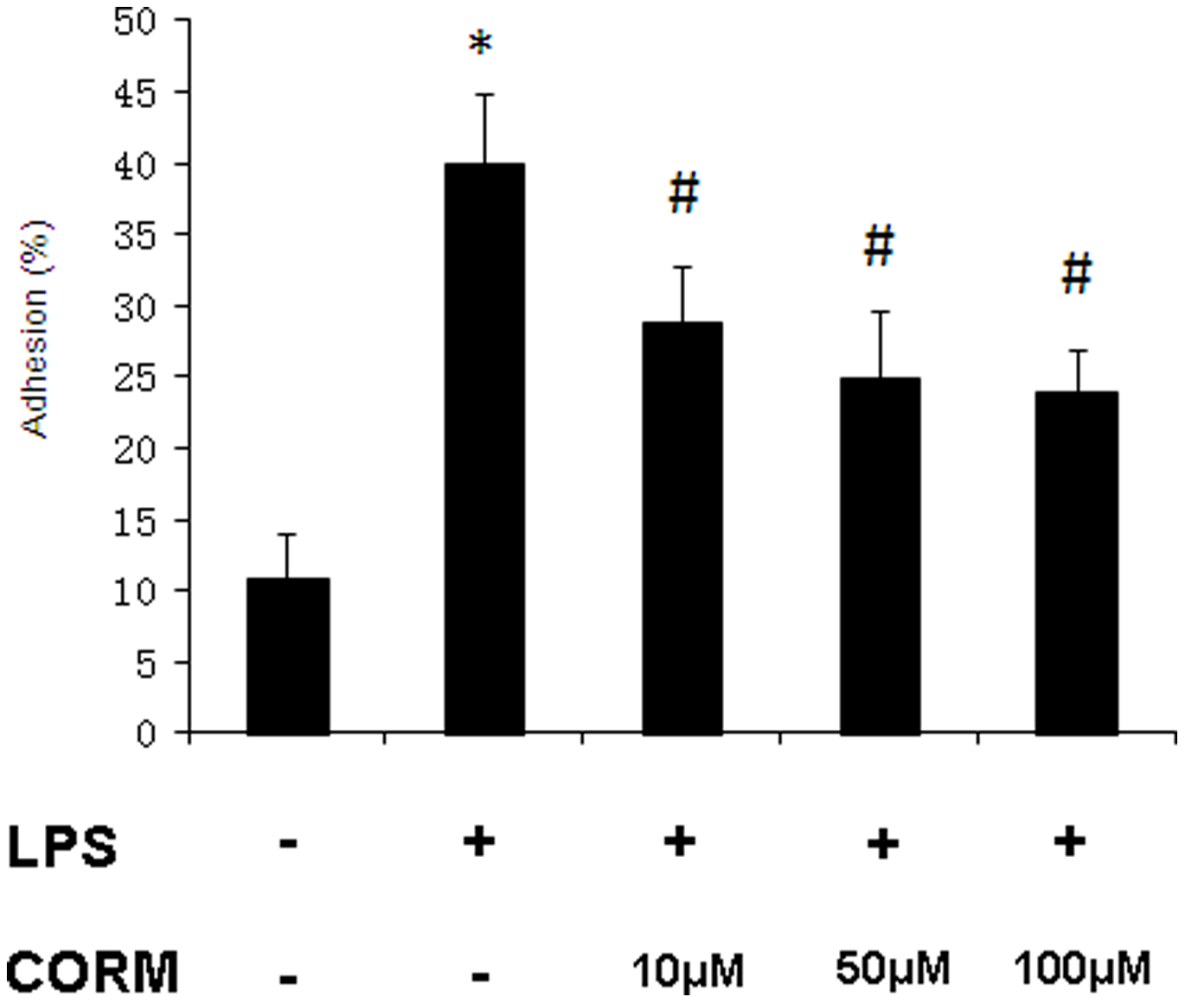
Effect of CORM-2 on PMN adhesion to LPS-stimulated HUVECs. Confluent monolayers of HUVECs were incubated with LPS (10 µg/ml) in the presence or absence of CORM-2 (10, 50 and 100 µM) for 4 h followed by PMN addition in the well. PMN adherence was determined. All values are expressed as means ± SE (*n* = 3). *P<0.01 compared to control; #*P*<0.05 compared to LPS.

As shown in Figure 9, adhesion of PMN to HUVECs is low in control. After monolayer of endothelial cells were stimulated by LPS for 4 h, adhesion of PMN to HUVECs significantly increased (P<0.01 compared to control). However, co-incubation with LPS and CORM-2 (10, 50 and 100 µM) markedly decreased leukocyte–endothelial cells adhesion (P<0.05 compared to LPS group).

## Discussion

Sepsis is a common and serious medical condition caused by a severe systemic infection leading to a systemic inflammatory response, which frequently occurs after hemorrhage, trauma, burn, or abdominal surgery. It is a leading cause of morbidity and mortality in severely ill patients [39]–[41]. Although some information has been generated from the LPS injection studies, LPS injection is an adequate model of endotoxemia and can not precisely mimic the changes observed during sepsis. On the other hand, cecal ligation and puncture (CLP) model seems to resemble qualitatively as well as quantitatively the clinical observations of vascular reactivity and inflammation during polymicrobial peritonitis, bacteremia, and systemic sepsis [42]. Therefore, the aim of this study is to evaluate the possible role of CORM-derived CO in CLP-induced sepsis.

Many experimental studies have highlighted the specific and independent role of exogenous CO in the modulation of inflammation [43], [44]. As new metal carbonyl-based compounds, CO-releasing moleculars (CORMs) have the ability to release CO in biological systems. The vasoactive, anti-hypertensive and anti-rejection effects of CO-RMs have been demonstrated to be due to the CO liberated by the compounds. CORM-2 also has exhibited anti-inflammatory actions in an *in vitro* model of LPS-stimulated macrophages [45]. Therefore, the aim of this study was to investigate the effects of CORM-2 on the dynamics of leukocytes sequestration and inflammatory responses in the lung and liver, and on the decrease of ROS and NO production in the LPS-stimulated HUVECs.

Pro-inflammatory cytokines, such as TNF-α and IL-6 have been shown to be released early after an inflammatory stimulus. To confirm if suppressing inflammatory responses in sepsis by CORM-2 was partly through interruption of the cycle of inflammatory events, we investigated the expression of inflammatory cytokines TNF-α and IL-6 in serum of the septic mice. We observed marked increases in TNF-α and IL-6 levels in serum after CLP injury. *In vitro* administration of CORM-2 was able to inhibit the inflammatory production induced by CLP. Our findings strongly indicated that CORM-2 appears to inhibit upregulation of inflammatory production, and consequently might effectively decrease inflammatory response induced by CLP.

Leukocytes sequestration and their subsequent infiltration in lung and liver tissues can cause leukocyte activation and contribute to vascular damage and the development of systemic inflammatory reaction. Myeloperoxidase (MPO) is an enzyme that is found predominantly in the azurophilic granules of polymorphonuclear leukocytes (PMN). Tissue MPO activity is frequently utilized to estimate tissue PMN accumulation in inflamed tissues and correlates significantly with the number of PMN determined histochemically in tissues [46], [47]. In the present study, we found that tissue MPO activity was markedly elevated after CLP and *in vivo* administration of CORM-2 led to the significantly downregulation of MPO activity. This indicated that CORM-2 effectively prevents PMN chemotaxis and infiltration in the tissue after CLP, consequently decreased the production of oxidants, reduced tissue oxidative injury.

Previously, we demonstrated that thermal injury induced lung and liver neutrophil deposition, organ iNOS expression [28]. We have also shown that NO from iNOS regulated proinflammatory activation, gene expression, and tissue injury in the liver after thermal injury, and CORM-2 inhibited the expression of iNOS in liver tissues, reducing liver injury and tissue PMN infiltration in thermally injured mice [29]. In this study, we measured expression levels of iNOS in the tissues of CLP mice. We found that there was a significant increase of tissue iNOS activities, suggesting that peroxynitrite plays a vital role in CLP-induced organ damage. The expressions of iNOS were significantly inhibited by in vivo administration of CORM-2. In parallel, the in vitro experiments showed that LPS caused a significant increase of Oxidant production and NO production in HUVECs, which was prevented effectively with CORM-2 treatment. These data showed that CORM-2 exhibits, at least partly, an important role in inhibiting iNOS expression, subsequently downregulating the NO production, and attenuating the oxidative stress and tissue damage.

The direct cause of leukocytes sequestration after CLP is considered to be the more expression of adhesion molecule (ICAM-1). ICAM-1 activates leukocytes and endothelial cells (ECs), which in turn prompt the release of various inflammatory mediators, resulting in systemic inflammatory response syndrome (SIRS), acute respiratory distress syndrome (ARDS) and multi-organ dysfunction syndrome (MODS)[48]–[51]. The present results showed that CORM-2 inhibits the increase of ICAM-1 expression in LPS-stimulated HUVECs. In addition, the results of *in vitro* experiments showed that LPS stimulation caused significant increase of PMNs adhesion to HUVECs, CORM-2 treatment effectively prevented this increase.

There is no doubt that the nuclear factor (NF-κB) is a ubiquitous, rapidly acting transcription factor involved in immune and inflammatory reactions, it exerts its immune and inflammatory response by regulating expression of cytokines, chemokines, cell adhesion molecules, and growth factors [52]–[54]. In this study, NF-κB activities in lung and liver tissue in septic mice and in LPS-stimulated HUVECs were elevated while they were markedly inhibited by administration of CORM-2. These data showed that CORM-2 plays a pivotal role in inhibition of NF-κB activity, subsequently decreased the expression of cellular adhesion molecules (ICAM-1) and CLP-induced pro-inflammatory mediators. Therefore, an effective therapeutic strategy that inhibits this transcription factor would be expected to improve organ functions during sepsis.

In summary, the present studies serve to clarify the role of CORM-2, one of the novel CO-releasing molecules, on the mechanisms of attenuation of leukocyte sequestration and inflammatory responses during sepsis. Application of CORM-2 on CLP mice attenuated PMNs accumulation, prevented activation of NF-κB, and subsequently decreased the production of inflammatory mediators in the lung and liver. This was accompanied by a downregulation of the expression of iNOS. In parallel, expression of ICAM-1, PMN adhesion to HUVECs stimulated by LPS were markedly decreased after CORM-2-treatment. Taken together these findings indicate that CORM-released CO attenuates inflammatory responses by interfering with NF-κB activation and protein expression of iNOS and ICAM-1.
